# CXCR4, the master regulator of neutrophil trafficking in homeostasis and disease

**DOI:** 10.1111/eci.12949

**Published:** 2018-05-23

**Authors:** Katia De Filippo, Sara M. Rankin

**Affiliations:** ^1^ IRD Section Respiratory Division NHLI Faculty of Medicine Imperial College London London UK

**Keywords:** clearance, CXCR4, mobilization, neutrophils, retention

## Abstract

**Background:**

Chemokines play a critical role in orchestrating the distribution and trafficking of neutrophils in homeostasis and disease.

**Results:**

The CXCR4/CXCL12 chemokine axis has been identified as a central regulator of these processes.

**Conclusion:**

In this review, we focus on the role of CXCR4/CXCL12 chemokine axis in regulating neutrophil release from the bone marrow and the trafficking of senescent neutrophils back to the bone marrow for clearance under homeostasis and disease. We also discuss the role of CXCR4 in fine‐tuning neutrophil responses in the context of inflammation.

## 1 CXCR4

CXCL12 was first identified as a chemoattractant for lymphocytes produced by cultures of bone marrow stromal cells—hence its original name, stromal‐derived factor‐1 (SDF‐1).^1^ CXCR4 was later identified as the receptor for CXCL12 and for many years was thought to be its sole receptor.^2^ Initial studies showed that genetic deletion of CXCR4 in mice was embryonically lethal and prevented the trafficking of haematopoietic stem cells from the yolk sac to the bone marrow, indicating a critical role for CXCR4 in development and the establishment of the haematopoietic system in the bone marrow.^3^ Later work identified CXCR4 as a key regulator in development in the context of zebrafish.^4^ CXCR4 expressed by lymphocytes was shown to play a critical role in their trafficking under homoeostatic conditions, but of potentially important therapeutic relevance, CXCR4 was identified as one of the chemokine receptors hijacked by HIV for entry into lymphocytes.^5^ This finding prompted work to develop specific CXCR4 antagonists. The first such antagonist to be taken into humans was AMD3100, or Plerixafor.

## 2 CXCL12

From the original tissue expression studies, the bone marrow was identified as a key source of CXCL12 under homeostasis in mice and man; however, its function was unknown.^1^ G‐CSF is a granulocyte colony‐stimulating factor, and when administered to mice or humans stimulates a significant increase in circulating neutrophil numbers. Elegant work from Daniel Link's laboratory showed that this was due not only to the ability of G‐CSF to stimulate neutrophil proliferation and maturation in the bone marrow, but also to stimulate the egress of neutrophils from the bone marrow into the blood.^6^ In a seminal paper Link determined that G‐CSF in mice acted indirectly to stimulate neutrophil mobilisation and noted a reduction in CXCL12 mRNA in the bone marrow following G‐CSF administration.^6^ This was the first link between CXCL12 levels in the bone marrow and neutrophil numbers in the blood; however, it was unclear what the mechanistic link was, as in the original studies it was reported that neutrophils did not migrate in response to CXCL12.^1^

## 3 CXCR4 AND NEUTROPHIL RETENTION IN THE BONE MARROW

A large storage pool of mature neutrophils is retained within the haematopoietic compartment of the bone marrow for up to 4‐6 days, constituting the bone marrow reserve both in humans and in mice. In mouse, it is estimated that the ratio between bone marrow and blood for mature neutrophils is 300:1. This reflects the known fact that neutrophils can be rapidly mobilised, increasing circulating levels in under an hour, for example in response to tissue injury or infection, as discussed in more detail below. The mechanisms underlying the retention of mature neutrophils in the bone marrow were unknown for many years.

**Figure 1:**
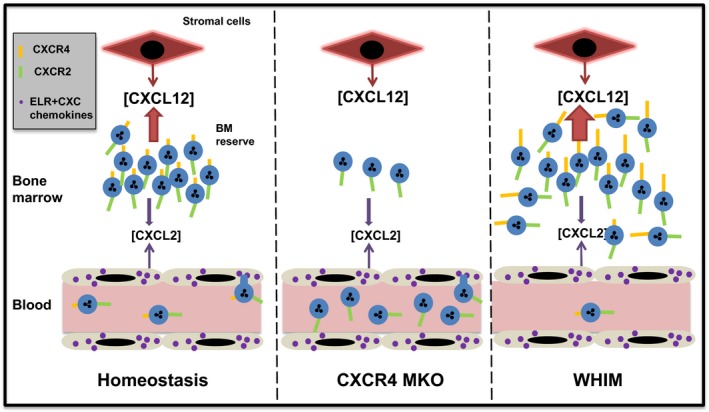
The CXCL12/CXCR4 neutrophil retention axis. There is a substantial storage pool of mature neutrophils in the bone marrow termed the bone marrow reserve. Under homeostasis, neutrophils are retained in the bone marrow due to the constitutive production of CXCL12 by stromal cells and expression of CXCR4 by neutrophils. Genetic deletion of CXCR4 in myeloid cells (CXCR4MKO) results in depletion of the bone marrow reserve with a rise in circulating neutrophil numbers. In WHIM syndrome, a genetic mutation of CXCR4 increases the activity of the receptor, thereby enhancing neutrophil retention in the bone marrow and reducing numbers in the blood

**Figure 2:**
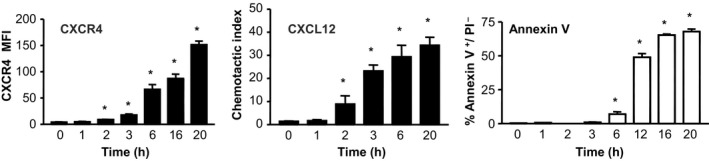
Upregulation of CXCR4 on human blood neutrophils in CXCL12‐free medium occurs rapidly, preceding apoptosis. Human neutrophils isolated from the blood of healthy volunteers were placed in culture in CXCL12‐free medium at 37°C and 5%CO_2_. Levels of CXCR4 annexin V and PI were determined by flow cytometry, and migration to CXCL12 was assessed in a transwell chemotaxis assay (unpublished data courtesy of Coralie Martin and Andrew Scourfield). *P < .05

AMD3100 is a selective CXCR4‐competitive antagonist. While originally developed as an HIV antagonist, phase‐I clinical studies indicated that this drug causes a rapid rise in circulating leucocytes, the majority of which were neutrophils.^7^, ^8^, ^9^, ^10^ In mice, AMD3100 was shown to stimulate an increase in circulating neutrophil numbers within a matter of hours, following ip injection.^11^ Subsequent studies performed by cannulating the murine femoral artery and vein in situ, such that the femoral bone marrow could be selectively perfused, showed directly that infusion of the CXCR4 antagonist led to the mobilisation of mature neutrophils and HSCs from the bone marrow into the vasculature.^11^, ^12^ This suggested that the CXCL12:CXCR4 axis was involved in the retention of mature neutrophils in the bone marrow (Figure 1). This was initially surprising, because neutrophils were not thought to express CXCR4. However, our studies revealed that neutrophils freshly isolated from the mouse bone marrow exhibited high intracellular levels of CXCR4.^11^ Moreover, when placed in medium lacking CXCL12, they quickly upregulated CXCR4 levels within a matter of hours.^11^ CXCR4, like other chemokine receptors, is internalised following ligand binding and receptor activation. Our interpretation of these observations is that the low levels of CXCR4 on the cell surface of neutrophils in the bone marrow, or those freshly isolated from the bone marrow, may be the result of down‐regulation of the receptor due to its continuous activation by high basal levels of CXCL12 produced locally in the bone marrow. Therefore, neutrophils in the bone marrow receive a retention signal. Further studies in human neutrophils showed a rapid upregulation of CXCR4 within a matter of hours when placed in CXCL12‐free medium (Figure 2). These data also show that neutrophils can respond robustly to CXCL12 when they express very low levels of CXCR4.

## 4 GENETIC MUTATION OF CXCR4 AFFECTING NEUTROPHIL RETENTION IN THE BONE MARROW

Mice with a myeloid lineage‐restricted deletion of CXCR4 (MKO), present with a neutrophilia in peripheral blood and a marked increase in the splenic pool of neutrophils, consistent with the concept that the CXCR4/CXCL12 axis is critical for neutrophil retention in the bone marrow (Figure 1).^13^ The fact that MKO mice fail to mobilise neutrophils in response to G‐CSF or KC has been interpreted as providing evidence that neutrophil release from bone marrow is dependent on CXCR4 signalling.^13^ However, 1 caveat of this interpretation is that the neutrophil reserve in the bone marrow of the MKO mice is severely depleted (0.6 × 10^7^neutrophils/femur in the MKO mice as compared to 1.2 × 10^7^ neutrophils/femur in the WT) which will naturally limit numbers that can be mobilised.

Recently, studies of neutrophil trafficking in zebra fish showed that mutation of CXCR4 or CXCL12 led to an increase in circulating neutrophils with a concomitant reduction in numbers in the caudal hematopoietic tissue, effectively recapitulating the data in mice.^14^

## 5 WHIM SYNDROME AND ABNORMAL NEUTROPHIL RETENTION

WHIM syndrome is an autosomal dominant human disorder caused by heterozygous mutations in the gene coding for CXCR4. WHIM‐associated mutations of CXCR4 result in the production of a C‐terminal truncated receptor that displays impaired internalisation and enhanced signalling, resulting in abnormal neutrophil retention in the bone marrow (myelokathexis) and a consequent chronic blood neutropenia (Figure 1).^15^, ^16^ Therefore, despite having normal numbers of mature neutrophils in the bone marrow, some WHIM patients exhibit a blood neutropenia. This severe neutropenia causes recurrent respiratory bacterial infections.

Administration of G‐CSF to WHIM patients, by lowering CXCL12 levels in the bone marrow, is sufficient to cause an increase of circulating neutrophils lasting for hours after the treatment.^17^ Pharmacologic antagonism of CXCR4 represents a novel approach for the treatment of WHIM patients.^18^ Indeed, a single dose of AMD3100 can rapidly and transiently increase neutrophil numbers for 1‐9 hours and lasting up to 24 hours in some patients.^19^ This therapeutic use of AMD3100 in WHIM patients was shown to considerably reduce infection frequency and wart burden.^20^

## 6 MECHANISM OF ACTION OF AMD3100

**Figure 3:**
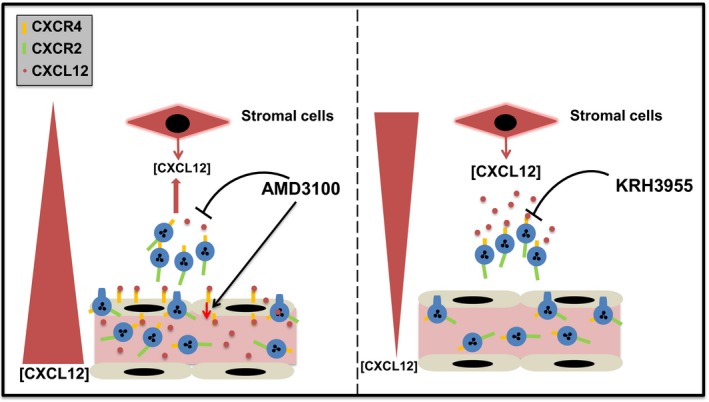
Neutrophil mobilisation by CXCR4 antagonists. Two CXCR4 antagonists AMD3100 and KRH3955, both stimulate neutrophil mobilisation into the blood, but by distinct mechanisms of action in vivo. AMD3100 interacts with the transmembrane domain of CXCR4 and stimulates the translocation of CXCL12 from the bone marrow stroma across the sinusoidal endothelium, increasing CXCL12 levels in the blood with a concomitant reduction in CXCL12 levels in the bone marrow. Neutrophils migrate in response to increased levels of CXCL12 in the blood. In contrast KRH3955 binds to the extracellular domain of CXCR4 and blocks binding of CXCL12 to the receptor, thereby directly disrupting the CXCL12/CXCR2 neutrophil retention axis in the bone marrow

It was originally thought that AMD3100 stimulated neutrophil mobilisation due to direct antagonism of CXCR4 expressed by neutrophils, thereby disrupting the CXCL12‐CXCR4 retention axis in the bone marrow. It was subsequently shown in mice that CXCR4 expressed by the bone marrow endothelium (BME) was involved in the translocation of CXCL12 across the BME, and that AMD3100 enhanced this process, resulting in a rapid rise of CXCL12 in the blood and reduced levels in the bone marrow.^21^ As such, it was proposed that by reversing the gradient of CXCL12 across the BME neutrophils would exit the bone marrow in a CXCR4‐dependent manner, migrating towards the highest concentration of CXCL12 in the blood.^21^, ^22^ To test this hypothesis, we made use of chalcone 4‐phosphate, a CXCL12‐neutralising ligand. Our studies revealed that chalcone 4‐phosphate, when administered alone, had no effect on circulating numbers of murine neutrophils; however, it significantly suppressed the mobilisation of neutrophils by AMD3100.^23^ This is consistent with a model whereby the AMD3100‐induced rise in circulating CXCL12 is critical for neutrophil mobilisation (Figure 3). In contrast, KRH3955, a distinct CXCR4 antagonist, does not reverse the CXCL12 gradient across the BME, but still efficiently mobilises neutrophils from the bone marrow, implying that KRH3955 disrupting the CXR4/CXCL12 retention axis directly is sufficient for mobilisation under homeostasis (Figure 3).^23^ To understand why these 2 CXCR4 antagonists have different physiological mechanisms of action, we need to consider how they interact with CXCR4 at the molecular level. CXCL12 normally binds to CXCR4 in a stepwise manner, with the β‐sheet and 50‐s loop of CXCL12 first interacting with the extracellular region of CXCR4 and secondly the N‐terminal domain of CXCL12 interacting with the transmembrane region of CXCR4.^24^ Studies of the molecular interaction of AMD3100 with CXCR4 predict that AMD3100 intercalates in the transmembrane region of CXCR4; thus, it is thought that CXCL12 can then still bind to the extracellular region of CXCR4.^21^, ^24^, ^25^, ^26^ What is not clear is the mechanism whereby the translocation of CXCL12 across the BME is enhanced due to the binding of AMD3100 in the transmembrane region of CXCR4. In contrast, KRH3955, a distinct CXCR4 antagonist, binds to the extracellular domain of CXCR4 and thus completely blocks CXCL12 interacting with CXCR4.^26^ Unlike AMD3100, KRH3955 does not reverse the CXCL12 gradient across the BME, but still mobilises neutrophils from the bone marrow, implying that in the case of KRH3955, directly disrupting the CXCL12/CXCR4 retention axis is sufficient to stimulate neutrophil mobilisation. These data indicate that there are alternative mechanisms underlying the ability of CXCR4 antagonists to stimulate neutrophil mobilisation from the bone marrow, dependent on their site of binding to CXCR4.

## 7 TISSUE ORIGIN OF NEUTROPHILS MOBILISED INTO THE BLOOD WITH AMD3100

In contrast to the original studies discussed above, in a manuscript published by Devi et al,^27^ data were presented showing that AMD3100 administration prompted the demargination of murine neutrophils from the lung microvascular bed and suggested that the observed increases in circulating neutrophil numbers following AMD3100 treatment were due to release from the lung microvasculature combined with an inhibition of aged neutrophils trafficking back to the bone marrow. Further in intravital microscopy IVM studies of the murine skull bone marrow, it was reported that administration of AMD3100 did not stimulate an observable mobilisation of neutrophils from the bone marrow.^27^ However, subsequent thorough analysis of neutrophil redistribution following AMD3100 administration did not support these findings.^26^ Thus, Liu Q. reported an increase as opposed to a decrease in numbers of murine neutrophils in the lung microvasculature following AMD3100 treatment and a decrease in bone marrow numbers, consistent with the original data showing that neutrophils were being mobilised from the BM by AMD3100.^11^, ^28^ More recently, IVM of the murine lung microvasculature has identified 2 distinct neutrophil migratory behaviours, with some neutrophils engaging with the endothelium and crawling, while others are simply transported with the flow of blood^29^ (K. De Filippo, unpublished observation). In our own studies in mice, we saw a similar pattern of neutrophil behaviour, and up to 2.5 hours following administration of AMD3100 we did not observe a decrease in number of neutrophils engaging with the endothelium. We also observed an increase in free‐flowing neutrophils in the lung microvasculature following AMD3100 administration, consistent with the results of Liu (K. De Filippo, unpublished observation). Likewise, in another independent recent report, AMD3100 was shown to increase activated tissue reverse transmigrated neutrophil numbers in the lung and reduce numbers in the bone marrow, in a model of mouse sterile liver injury.^30^ Thus, on balance, the data reported thus far support the hypothesis that AMD3100 increases the circulating number of neutrophils by stimulating their mobilisation from the bone marrow reserve, as opposed to demargination from the lung microvasculature.

## 8 THE POTENTIAL ROLE OF THE CXCR4/CXCL12 AXIS IN NEUTROPHIL RETENTION IN THE SPLEEN

After the bone marrow, murine spleen is reported to express the highest levels of CXCL12 under homeostasis.^31^ Whether CXCL12 expressed in the spleen plays any role in neutrophil trafficking or retention in this tissue is currently relatively unexplored. However, it has been shown that numbers of neutrophils in the spleen are not reduced following AMD3100 administration, suggesting that CXCL12 expressed in murine spleen is not regulating neutrophil retention in this tissue.^28^ Moreover, AMD3100‐induced mobilisation of neutrophils was not suppressed in splenectomised mice; indeed, the rise in circulating neutrophils by AMD3100 was almost 3‐fold higher in splenectomised mice.^28^ Consistent with this finding is our observation that when murine neutrophil trafficking in the spleen is observed by IVM, AMD3100 administration substantially increased the number of neutrophils observed trafficking through the spleen (K. de Filippo, unpublished observation). These current data thus suggest that neutrophils are not actively retained in the spleen via CXCL12/CXCR4.

## 9 CXCR4 AND NEUTROPHIL CLEARANCE

Neutrophils are short‐lived cells with an estimated half‐life of 6‐12 hours in mice. They are cleared from the blood under homeostasis via liver, spleen and bone marrow.^32^ Apoptotic neutrophils are phagocytosed by macrophages, and the presence of macrophages in the reticuloendothelial system would, in theory, allow for the direct clearance of apoptotic neutrophils from the circulation. In contrast, clearance via macrophages in the bone marrow would require aged neutrophils to transmigrate across the bone marrow endothelium. An upregulation of CXCR4 was originally reported on human neutrophils after ageing in culture for 20 hours, and it was shown that aged neutrophils could migrate towards CXCL12.^33^ In contrast, others have reported that CXCR4 expression is only observed on apoptotic human neutrophils and that these neutrophils do not migrate in response to CXCL12. Our studies show that functional levels of CXCR4 are expressed on murine and human neutrophils incubated ex vivo within a matter of hours^11^ (Figure 2). Importantly, upregulation of functional levels of CXCR4 preceded enhanced binding of annexin V, a marker of neutrophil apoptosis (Figure 2). In our studies, such CXCR4^hi^ annexin V^−^ neutrophils could migrate in response to CXCL12, while neutrophils aged overnight that became CXCR4^hi^ annexin V^+^ apoptotic neutrophils did not migrate to CXCL12.^34^

**Figure 4:**
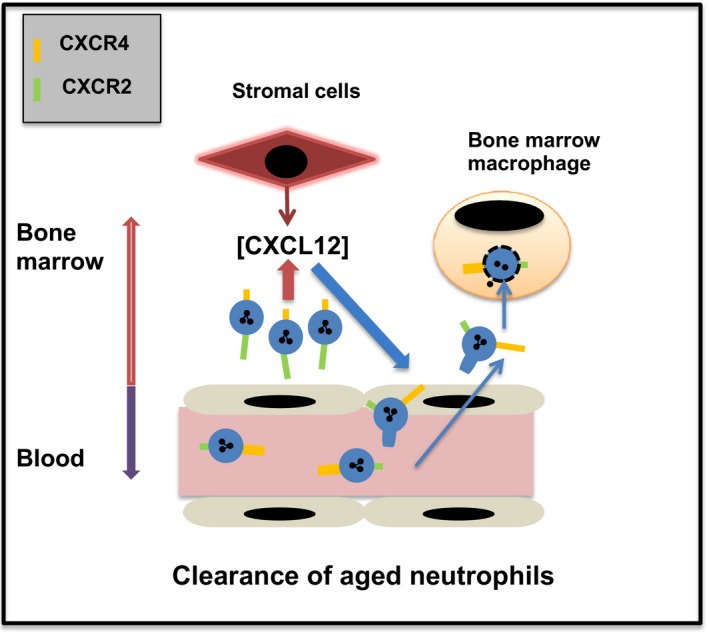
CXCR4‐dependent neutrophil clearance in the bone marrow upregulation of CXCR4 on neutrophils as they age increases their trafficking back to the bone marrow, where they are phagocytosed by bone marrow macrophages

In vivo experiments showed that CXCR4^hi^ aged murine neutrophils preferentially trafficked back to the bone marrow in a CXCR4‐dependent manner (Figure 4).^11^ Further studies with ^111^In labelled neutrophils allowed for quantification of absolute numbers of neutrophils cleared by different tissues and showed that the bone marrow, liver and spleen contributed equally to murine neutrophil clearance under homoeostatic conditions.^35^ A caveat of these studies was that the neutrophils were aged in vitro. That said, work by Casanova‐Acebes demonstrated the presence of a subpopulation of aged CXCR4^hi^CD62L^lo^ murine neutrophils in the blood.^36^ CXCL12 levels in the bone marrow follow a circadian rhythm, regulated by the sympathetic nervous system.^37^ A recent murine study has shown that senescent CXCR4^hi^ neutrophils are cleared from the blood when CXCL12 levels peak in the bone marrow.^36^ However, the studies by Casanova‐Acebes indicated that CXCR4 did not guide neutrophils back to the bone marrow but affected their subsequent anatomic localisation within the marrow tissue.^36^ In contrast, in an elegant study, Wang et al^30^ generated a genetically modified mouse in which the Ly6G+ neutrophils expressed photoactivable GFP. Sterile injury of the liver resulted in the recruitment of large numbers of neutrophils to the site of injury. These neutrophils at the injury site were photoactivated to investigate their site of clearance. Surprisingly, contra to current dogma that predicts clearance locally by tissue macrophages, the majority of GFP+ neutrophils were observed re‐entering the vasculature and subsequently trafficking selectively back to the bone marrow for clearance.^30^ Finally, it was shown that the GFP+ neutrophils were CXCR4^hi^, and trafficking back to the bone marrow was blocked by the CXCR4 antagonist AMD3100.^30^, ^36^ It is interesting that the bone marrow is the sole site of neutrophil destruction in this context, and the pathophysiological reason for this observation needs further investigation. Taken together, these studies suggest a role for CXCR4 in neutrophil clearance under homoeostatic condition and following trauma. Of note, while there is now considerable evidence for a CXCR4‐dependent pathway of clearance for neutrophils in the bone marrow in mice, it is currently not known whether a similar pathway operates in man.

## 10 CXCR2/CXCR4 INTERPLAY IN INFLAMMATION

**Figure 5:**
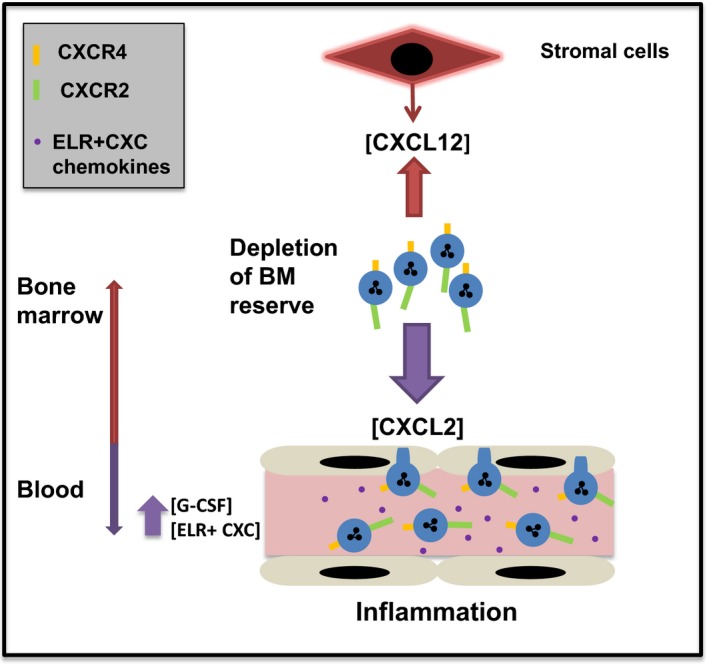
In response to inflammation, an increase in circulating GCSF and ELR+ CXC chemokines drives neutrophil egress from the bone marrow reserve into the blood resulting in a rapid rise in circulating numbers of neutrophils

Under inflammatory conditions, both the mobilisation and recruitment into inflamed tissue are orchestrated by an interplay between Glu‐Leu‐Arg tripeptide‐containing (ELR^+^) CXC chemokines and CXCL12.^38^, ^39^, ^40^ Studies have shown that chemokines and G‐CSF generated remotely at sites of inflammation act systemically to stimulate murine neutrophil mobilisation from the bone marrow.^41^, ^42^ In this context, the mobilisation of neutrophils by ELR^+^ CXC chemokines is tempered by the CXCL12/CXCR4 retention axis (Figure 5). This has been shown directly by the fact that CXCL2 and the CXCR4 antagonist AMD3100 act synergistically to mobilise neutrophils from the bone marrow,^11^ while G‐CSF mobilisation of neutrophils has been shown to be CXCR2 dependent, with G‐CSF stimulating the release of CXCL2 from the murine bone marrow endothelium.^43^, ^44^

At the site of infection/inflammation, CXCR4 plays a more nuanced role in modulating the immune response. Thus, in the context of the lung, 1 study in mice reported that CXCR4 is important in migration of neutrophils from within the lung tissue across the epithelium into the alveolar spaces.^45^ Another study in mice showed that a subpopulation of CXCR4^hi^ neutrophils constituted the first line of defence by rapid migration to the site of inflammation during an acute inflammatory response.^46^ Further evidence was presented that these “experienced” neutrophils were able to phagocytose pathogens more efficiently compared with fresh, mature neutrophils.^46^ The fact that surface expression of CXCR4 on aged human neutrophils can be downregulated by stimuli such as IFN‐α, IFN‐γ, GM‐CSF and G‐CSF at the mRNA level suggests that there is scope for neutrophil responses to CXCL12 to be further fine‐tuned at the site of inflammation.^33^

## 11 HMGB‐1 AND CXCR4

During sterile inflammation or tissue trauma, damage‐associated molecular patterns (DAMPs) are actively or passively secreted by damaged cells to inform the immune system of tissue injury or damage. HMGB1 (High Mobility Group Box 1) is a prototypical DAMP released by the nucleus of necrotic cells.^47^ HMGB‐1 is a DAMP generated at sites of tissue damage. Studies in humans and mice have shown that HMGB‐1 interacts directly with CXCL12, and the resulting complex binds exclusively to CXCR4; the complex has enhanced potency as compared to CXCL12 alone.^48^ Moreover, by conditional ablation strategies in mice, it has been shown that epithelial HMGB1 triggers specific recruitment of neutrophils, but not macrophages, towards necrotic tissue.^49^

## 12 EPI‐X4 AN ENDOGENOUS CXCR4 ANTAGONIST

Recently, an endogenous peptide EPI‐X4 has been identified that is generated by proteolysis of serum albumin by cathepsins at acidic pH. EPI‐X4 has been shown to be a selective and potent CXCR4 antagonist.^50^ When administered ip in mice, it stimulates an increase in circulating neutrophils, much like AMD3100. It has been proposed that this peptide could be generated at sites of inflammation, where the microenvironment is known to be acidic. Its function in this context is unknown, but one possibility is that it allows CXCR4^hi^ neutrophils at sites of inflammation to reverse‐migrate back into the circulation, by reducing their retention in the tissue.

## 13 CONCLUSION

From original studies suggesting that neutrophils did not express CXCR4 and did not migrate in response to SDF, work over the last 16 years has revealed a critical role for the CXCL12/CXCR4 chemokine axis in neutrophil biology. CXCR4 has been shown to be a master regulator of neutrophil retention in the bone marrow and mobilisation during inflammatory responses in mouse at least and unexpectedly also in neutrophil clearance. Moreover, the identification of human CXCR4 mutations in WHIM patients has led to the use of a CXCR4 antagonist, originally developed as an HIV antagonist, for treatment of these neutropenic patients as an alternative to G‐CSF treatment.
